# SDHB and SDHA Immunohistochemistry in Canine Pheochromocytomas

**DOI:** 10.3390/ani10091683

**Published:** 2020-09-17

**Authors:** Firas M. Abed, Melissa A. Brown, Omar A. Al-Mahmood, Michael J. Dark

**Affiliations:** 1 Department of Comparative, Diagnostic and Population Medicine, College of Veterinary Medicine, University of Florida, Gainesville, FL 32610, USA; fabed@ufl.edu; 2 Veterinary Diagnostic Laboratories, College of Veterinary Medicine, University of Florida, Gainesville, FL 32610, USA; melissabrown@ufl.edu; 3 Department of Food, Nutrition and Packaging Science, College of Agriculture, Forestry and Life Sciences, Clemson University, Clemson, SC 29634, USA; oabdull@g.clemson.edu; 4 Emerging Pathogens Institute, University of Florida, Gainesville, FL 32610, USA

**Keywords:** dog, pheochromocytoma, SDH, Immunohistochemistry

## Abstract

**Simple Summary:**

Pheochromocytomas are adrenal tumors that occur in both dogs and people. One of the more common gene families involved in the development of this tumor in people is succinate dehydrogenase (SDH). In people, immunohistochemistry can be used with biopsy samples to predict gene pathways that may be involved in the development of the tumor. This is faster and cheaper than performing extensive sequencing to determine if genes are involved. We tested 35 dog tumors to determine how likely SDH mutations were. While our data suggest significant numbers of SDH mutations, these mutations do not appear to be associated with tumor aggression.

**Abstract:**

Pheochromocytomas (PCs) are tumors arising from the chromaffin cells of the adrenal glands and are the most common tumors of the adrenal medulla in animals. In people, these are highly correlated to inherited gene mutations in the succinate dehydrogenase (SDH) pathway; however, to date, little work has been done on the genetic basis of these tumors in animals. In humans, immunohistochemistry has proven valuable as a screening technique for SDH mutations. Human PCs that lack succinate dehydrogenase B (SDHB) immunoreactivity have a high rate of mutation in the SDH family of genes, while human PCs lacking succinate dehydrogenase A (SDHA) immunoreactivity have mutations in the SDHA gene. To determine if these results are similar for dogs, we performed SDHA and SDHB immunohistochemistry on 35 canine formalin-fixed, paraffin-embedded (FFPE) PCs. Interestingly, there was a loss of immunoreactivity for both SDHA and SDHB in four samples (11%), suggesting a mutation in SDHx including SDHA. An additional 25 (71%) lacked immunoreactivity for SDHB, while retaining SDHA immunoreactivity. These data suggest that 29 out of the 35 (82%) may have an SDH family mutation other than SDHA. Further work is needed to determine if canine SDH immunohistochemistry on PCs correlates to genetic mutations that are similar to human PCs.

## 1. Introduction

Pheochromocytomas (PCs) are catecholamine-secreting neuroendocrine tumors arising from the chromaffin cells of the neural crest [1,2,3]. PCs are more often seen in dogs and cattle [4] and can be unilateral or bilateral and functional or nonfunctional. While canine pheochromocytomas are usually benign, they can invade adjacent tissues and may be malignant, with metastasis to distant tissues [4]. The behavior of pheochromocytomas is difficult to predict based on histologic findings [5,6].

Immunohistochemistry (IHC) data has found that canine and human PCs are highly similar, as neoplastic cells in both share the expression of numerous antigens, including S100, synaptophysin (SYN), chromogranin A (CGA), and substance P (SP) [7]. Known genetic mutations are involved in the pathogenesis of approximately 60% of human PCs. Frequently, these are associated with mutations in the succinate dehydrogenase (SDH) family of genes, with mutations in succinate dehydrogenase subunit B (SDHB) associated with a high likelihood of metastasis/malignancy [5]. The sequencing of multiple SDH genes in every pheochromocytoma is economically infeasible in veterinary medicine, making the determination of the genetic basis of PC in dogs uncertain.

Studies in human PCs have found that immunohistochemistry (IHC) is highly correlated with the SDH mutation status. For instance, all samples with mutations in SDH family genes lacked SDHB immunoreactivity [8]. Loss of SDHB protein expression has therefore been used for prognostication in human medicine; in one study, the relationship between the SDH genetic background and SDHB immunohistochemistry sensitivity and specificity in human pheochromocytomas was 94.23% [9].

In veterinary medicine, while a link has been established between brachephalic dogs and pheochromocytoma [2], there has not been a definitive familial inheritance pattern like that described in people. Humans and dogs have a similar structure of SDH family genes [10]. Some mutations have been found in SDH family genes, which may indicate that mutations in these genes may initiate oncogenesis in a similar way to people [2,11]. While IHC has previously been used in canine pheochromocytomas to verify the neuroendocrine origin [4,7], only one study to date has examined the SDH family status in canine pheochromocytomas [11]. IHC would be significantly more cost-effective and practical than sequencing for mutation detection. In addition, if IHC is associated with patterns in clinical parameters, such as recurrence, invasion, or metastasis, this could be a valuable adjunct to histopathology.

In this study, we examined the expression of SDHA and SDHB in canine pheochromocytomas and compared these with data on patient age, tumor size, and invasion, in order to determine the utility of SDH IHC in canine pheochromocytoma diagnostics.

## 2. Materials and Methods 

### 2.1. Approval

This study was approved by the University of Florida Institutional Animal Use and Care Committee (study #201710050).

### 2.2. Samples

A total of 35 pheochromocytomas plus 40 control tissues (20 sections from the heart and 20 sections from normal adrenal glands) were obtained from paraffin-embedded tissue blocks in the College of Veterinary Medicine Anatomic Pathology, University of Florida tissue archive. Representative sections of each tumor were evaluated by a board-certified veterinary pathologist (MJD) to confirm the diagnosis. Clinical records from all samples from patients of the UF Veterinary Medical Center were analyzed to determine mass size and invasion.

### 2.3. Immunohistochemistry

Sections from each block were cut at 4 µm and stained using commercially available antibodies (SDHA Mouse Monoclonal Antibody [2E3GC12FB2AE2, ThermoFisher Scientific, catalog #459200] at 1:100 dilution and SDHB Rabbit Polyclonal Antibody [ThermoFisher Scientific, catalog #PA5-23079] at 1:75 dilution). Staining was performed on a Leica Bond immunostainer per the manufacturer’s directions. Sections from 20 canine adrenal glands and 20 cardiac samples were used to verify the appropriate immunoreactivity of the antibody with canine antigens.

Pheochromocytoma samples from dogs were listed as positive when there was a granular intracytoplasmic immunoreactivity with a similar intensity as the internal positive controls (endothelial cells, sustentacular cells, and/or lymphocytes). Negative samples lacked immunoreactivity in the cells of the mass that showed immunoreactivity in internal positive controls [9].

### 2.4. Statistical Analysis

Descriptive statistics were performed using JMP Pro 12 software (SAS Institute Inc., Cary, NC, USA) 21. Descriptive statistics that were calculated included averages and percentiles. The frequencies of positive SDHA and SDHB immunoreactivity between different variables (invasion, sex, breed, age, and animal weight) were evaluated and compared using Chi-square tests. For all statistical analyses, *p* > 0.05 was considered significant.

## 3. Results

**Figure 1:**
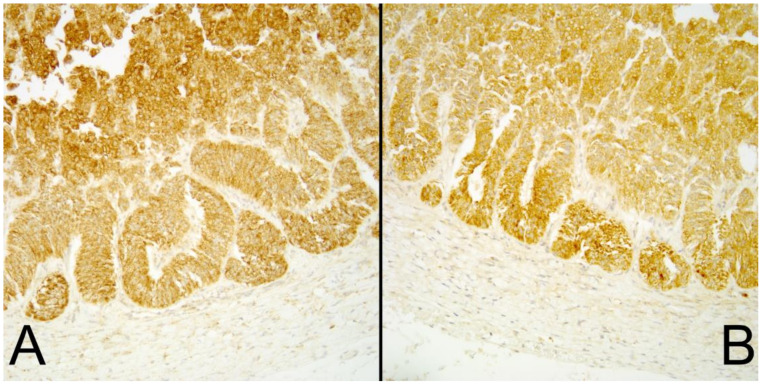
(**A**) Succinate dehydrogenase A (SDHA) and (**B**) Succinate dehydrogenase B (SDHB) immunoreactivity in normal canine adrenal glands as controls.

All 40 controls had an intracytoplasmic immunoreactivity for SDHA and SDHB similar to that found in human tissues (Figure 1A,B).

**Figure 2:**
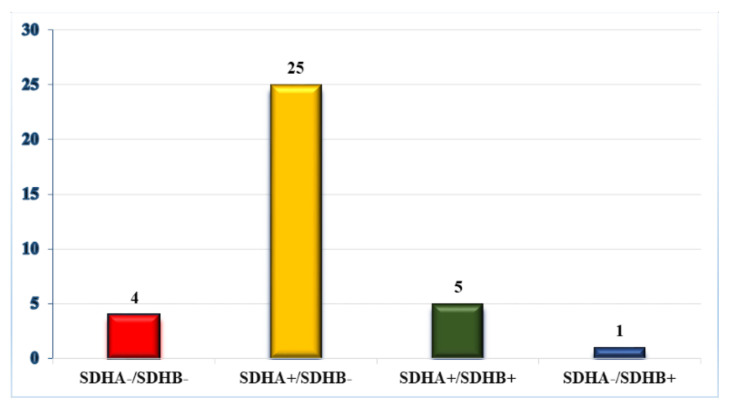
Succinate dehydrogenase A (SDHA)/ Succinate dehydrogenase B (SDHB) immunoreactivity for all samples.

**Figure 3:**
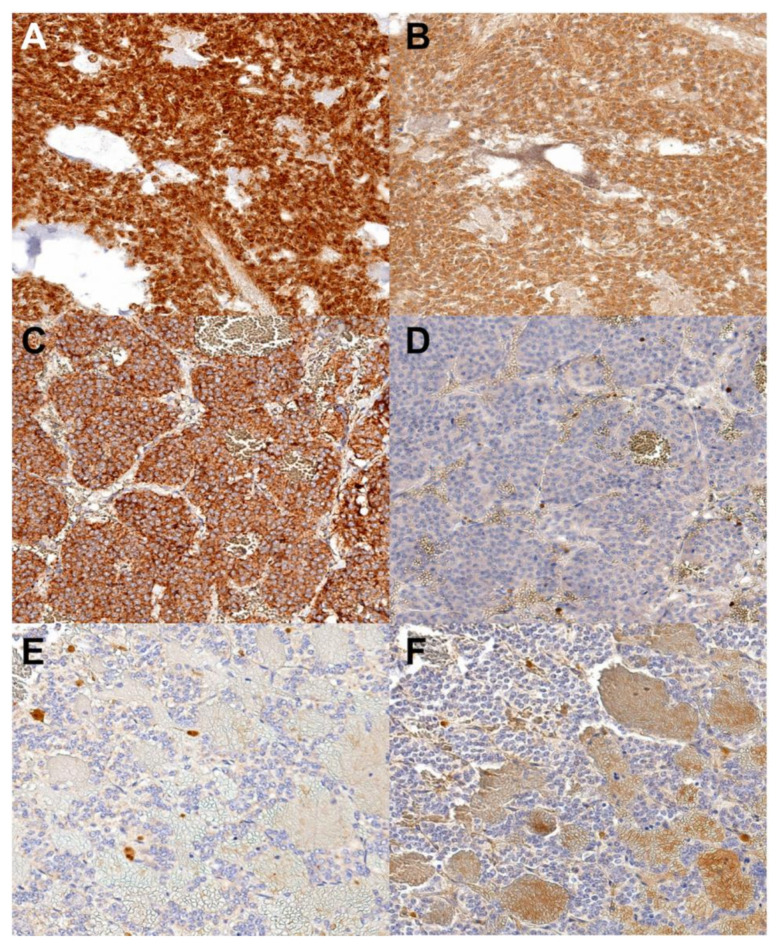
SDHA and SDHB immunoreactivity. (**A**,**C**,**E**)—SDHA immunohistochemistry. (**B**,**D**,**F**)—SDHB immunoreactivity. (**A**) and (**B**) represent a case with both SDHA and SDHB immunoreactivity; (**C**) and (**D**) have SDHA but lack SDHB immunoreactivity; (**E**) and (**F**) represent a case lacking both SDHA and SDHB immunoreactivity. SDHA—Succinate dehydrogenase A, SDHB—Succinate dehydrogenase B, Original objective 40×.

In the 35 sections examined, there was a lack of both SDHA and SDHB immunoreactivity in four samples (11.4%) (Figure 2 and Figure 3); 25 samples (71.4%) lacked SDHB immunoreactivity but had SDHA immunoreactivity. The lack of SDHB immunoreactivity correlated with both age (with younger animals predisposed (≤10 years old: 62.07%,>10 years old: 37.93%; *p* < 0.0421) and sex (male: 75.86%, female: 24.14%; *p* < 0.05).

Out of the 34 cases with associated signalment and clinical information, 12 were from females and 24 were from males (Table 1). The mean patient age was 10.4 years (standard deviation: 2.5 years). Out of 23 samples that had a clinical evidence of invasion, 19 (82.6%) lacked SDHB immunoreactivity. Of these, 14 samples (73.6%) had a vascular invasion (caudal vena cava, phrenicoabdominal vein, and/or renal vein), while two out of these 19 (10.53%) metastasized to the renal vein or regional lymph node. Out of the 12 samples with no reported invasion, 10 (34.84%) lacked immunoreactivity to SDHB. However, we lacked an intraoperative surgical report for four of these samples, which means that these may have an invasion that was not stated in the sample submission form. Two samples out of these 12 described adhesion to the omentum, splenic vessels, apex of the left pancreatic limb, and/or the left kidney. Out of the five samples that had both SDHA and SDHB immunoreactivity, three (60%) also had an invasion. There was no significant correlation between invasions and the immunohistochemical status, age, sex, breed, or animal weight.

## 4. Discussion

Human studies have found that 75% of samples with SDHA mutations lacked immunoreactivity for SDHA and SDHB [9] and that 90% of samples with SDHB, C, D, or SDHAF2 mutations had immunoreactivity for SDHA and lacked immunoreactivity for SDHB [9,12,13]. When this is applied to the samples in our study, these patterns suggest that 29 out of the 35 (82.8%) samples have a mutation in at least one of the SDH family genes (Figure 2).

The predominance of samples with an invasion lacking SDHB immunoreactivity agrees with several human studies [5,12,14,15], which show that a lack of SDHB immunoreactivity is associated with more aggressive tumor behavior.

Only one sample lacked SDHA immunoreactivity but maintained SDHB immunoreactivity (Figure 1); this sample showed signs of invasion and metastasis to the renal vein. However, as inactivation of SDHA does not lead to tumorigenesis [16], this may represent a somatic hypermethylation of the promoter region [17], leading to an accumulation of succinate and later an inhibition of demethylase enzymes. This can lead to promoter hypermethylation and tumor suppressor gene inactivation [18]; in humans, this is known as Leigh syndrome.

The lack of significant association between the immunohistochemical findings and tissue invasion may be due to limited numbers of cases with both SDHA and SDHB immunoreactivity.

Ultimately, while these data are encouraging, sequencing is needed to fully determine the role of SDH family genes in pheochromocytoma in canine pheochromocytomas. These findings do suggest that IHC has a similar utility in narrowing candidate mutations in pheochromocytomas in dogs; given, in particular, the relatively higher cost of sequencing, restricting the set of possible candidate genes using immunohistochemistry would be helpful in future sequencing studies. However, determining the true utility of SDH immunohistochemistry would require sequencing information and additional case information; if immunohistochemistry is associated with specific mutations and/or mutations in specific genes are closely associated with clinical behavior, immunohistochemistry would become an important tool in case management.

## 5. Conclusions

Canine pheochromocytomas have similar immunohistochemical characteristics to those previously reported in human tumors, with approximately 82% showing immunohistochemical evidence of an SDH family mutation. However, based on a small number of cases, there does not appear to be a correlation with invasion and SDH family gene mutation status. Further sequencing work is needed to verify the IHC results and to determine the genetic basis of canine pheochromocytoma; a larger pool of cases is also needed to confirm a lack of association between invasion and SDH family mutation status.

## Figures and Tables

**Table 1:** Immunohistochemical findings and clinical information for all cases.

Case	SDHA	SDHB	Invasion	Age	Sex	Breed
1	**−**	**−**	Caudal vena cava	10	M	Mixed Breed
2	**−**	**−**	None	8	M	Carolina Dog
3	**−**	**−**	Caudal vena cava	14	M	Mixed Breed
4	**−**	**−**	Caudal vena cava	8	M	Rhodesian Ridgeback
5	+	+	None	11	M	Terrier, Yorkshire
6	+	+	Caudal vena cava	13	F	Terrier, Scottish
7	+	+	Caudal vena cava	14	F	Mixed Breed
8	+	+	Phrenicoabdominal vein	11	F	Cocker Spaniel
9	+	+	None	8	F	Bassett Hound
10	+	**−**	Caudal vena cava, left external iliac	9	M	Mixed Breed
11	+	**−**	Caudal vena cava	9	M	Australian Shepherd
12	+	**−**	None	11	F	Schnauzer, Miniature
13	+	**−**	Caudal vena cava, left renal vein	13	F	Mixed Breed
14	+	**−**	Caudal vena cava	8	F	Rhodesian Ridgeback
15	+	**−**	Adhered to the dorsal body wall	12	M	Mixed Breed
16	+	**−**	None	7	F	Spaniel, Boykin
17	+	**−**	Phrenicoabdominal vein	12	M	Retriever, Golden
18	+	**−**	Adhered to the left pancreatic limb and the left kidney	16	M	Mixed Breed
19	+	**−**	Caudal vena cava, phrenicoabdominal vein	9	F	Miniature Schnauzer
20	+	**−**	Caudal vena cava, phrenicoabdominal vein	12	M	Jack Russell Terrier
21	+	**−**	None	9	M	Australian Blue Heeler
22	+	**−**	Caudal vena cava	8.4	M	Shetland Sheepdog
23	+	**−**	None	8.5	M	Rhodesian Ridgeback
24	+	**−**	Caudal vena cava, phrenicoabdominal vein	8.1	F	Doberman Pinscher
25	+	**−**	Phrenicoabdominal vein	11.8	M	Beagle
26	+	**−**	Caudal vena cava	9.7	M	Fox terrier
27	+	**−**	None	13	M	Dachshund
28	+	**−**	Mesenteric lymph node metastasis	11	M	Terrier, Boston
29	**−**	+	Renal vein	15	F	Retriever, Golden
30	+	**−**	None	12	M	Dachshund, Miniature
31	+	**−**	None	10	M	Rottweiler
32	+	**−**	Phrenicoabdominal vein	7	M	Schnauzer, Standard
33	+	**−**	No clinical information provided			
34	+	**−**	Caudal vena cava	5	F	Mixed Breed
35	+	**−**	Caudal vena cava	11	M	Mixed Breed

SDHA—Succinate dehydrogenase A, SDHB—Succinate dehydrogenase B, M—Male, F—Female.

## References

[1] Comino-Mendez I., de Cubas A.A., Bernal C., Alvarez-Escola C., Sanchez-Malo C., Ramirez-Tortosa C.L., Pedrinaci S., Rapizzi E., Ercolino T., Bernini G. (2013). Tumoral EPAS1 (HIF2A) mutations explain sporadic pheochromocytoma and paraganglioma in the absence of erythrocytosis. Hum. Mol. Genet..

[2] Holt D.E., Henthorn P., Howell V.M., Robinson B.G., Benn D.E. (2014). Succinate dehydrogenase subunit D and succinate dehydrogenase subunit B mutation analysis in canine phaeochromocytoma and paraganglioma. J. Comp. Pathol.

[3] Wilzen A., Rehammar A., Muth A., Nilsson O., Tesan Tomic T., Wangberg B., Kristiansson E., Abel F. (2016). Malignant pheochromocytomas/paragangliomas harbor mutations in transport and cell adhesion genes. Int. J. Cancer.

[4] Barthez P.Y., Marks S.L., Woo J., Feldman E.C., Matteucci M. (1997). Pheochromocytoma in dogs: 61 cases (19841–995). J. Vet. Intern. Med..

[5] Blank A., Schmitt A.M., Korpershoek E., van Nederveen F., Rudolph T., Weber N., Strebel R.T., de Krijger R., Komminoth P., Perren A. (2010). SDHB loss predicts malignancy in pheochromocytomas/sympathethic paragangliomas, but not through hypoxia signalling. Endocr. Relat. Cancer.

[6] Thompson L.D. (2002). Pheochromocytoma of the Adrenal gland Scaled Score (PASS) to separate benign from malignant neoplasms: A clinicopathologic and immunophenotypic study of 100 cases. Am. J. Surg. Pathol..

[7] Sako T., Kitamura N., Kagawa Y., Hirayama K., Morita M., Kurosawa T., Yoshino T., Taniyama H. (2001). Immunohistochemical evaluation of a malignant phecochromocytoma in a wolfdog. Vet. Pathol..

[8] Menara M., Oudijk L., Badoual C., Bertherat J., Lepoutre-Lussey C., Amar L., Iturrioz X., Sibony M., Zinzindohoue F., de Krijger R. (2015). SDHD immunohistochemistry: A new tool to validate SDHx mutations in pheochromocytoma/paraganglioma. J. Clin. Endocrinol. Metab..

[9] Papathomas T.G., Oudijk L., Persu A., Gill A.J., van Nederveen F., Tischler A.S., Tissier F., Volante M., Matias-Guiu X., Smid M. (2015). SDHB/SDHA immunohistochemistry in pheochromocytomas and paragangliomas: A multicenter interobserver variation analysis using virtual microscopy: A Multinational Study of the European Network for the Study of Adrenal Tumors (ENS@T). Mod. Pathol..

[10] Rustin P., Munnich A., Rotig A. (2002). Succinate dehydrogenase and human diseases: New insights into a well-known enzyme. Eur. J. Hum. Genet..

[11] Korpershoek E., Dieduksman D., Grinwis G.C.M., Day M.J., Reusch C.E., Hilbe M., Fracassi F., Krol N.M.G., Uitterlinden A.G., de Klein A. (2019). Molecular Alterations in Dog Pheochromocytomas and Paragangliomas. Cancers.

[12] Gill A.J., Benn D.E., Chou A., Clarkson A., Muljono A., Meyer-Rochow G.Y., Richardson A.L., Sidhu S.B., Robinson B.G., Clifton-Bligh R.J. (2010). Immunohistochemistry for SDHB triages genetic testing of SDHB, SDHC, and SDHD in paraganglioma-pheochromocytoma syndromes. Hum. Pathol..

[13] Van Nederveen F.H., Gaal J., Favier J., Korpershoek E., Oldenburg R.A., de Bruyn E.M., Sleddens H.F., Derkx P., Riviere J., Dannenberg H. (2009). An immunohistochemical procedure to detect patients with paraganglioma and phaeochromocytoma with germline SDHB, SDHC, or SDHD gene mutations: A retrospective and prospective analysis. Lancet. Oncol..

[14] Casey R.T., Ascher D.B., Rattenberry E., Izatt L., Andrews K.A., Simpson H.L., Challis B., Park S.M., Bulusu V.R., Lalloo F. (2017). SDHA related tumorigenesis: A new case series and literature review for variant interpretation and pathogenicity. Mol. Genet. Genomic Med..

[15] Pinato D.J., Ramachandran R., Toussi S.T., Vergine M., Ngo N., Sharma R., Lloyd T., Meeran K., Palazzo F., Martin N. (2013). Immunohistochemical markers of the hypoxic response can identify malignancy in phaeochromocytomas and paragangliomas and optimize the detection of tumours with VHL germline mutations. Br. J. Cancer.

[16] Eng C., Kiuru M., Fernandez M.J., Aaltonen L.A. (2003). A role for mitochondrial enzymes in inherited neoplasia and beyond. Nat. Rev. Cancer.

[17] Haller F., Moskalev E.A., Faucz F.R., Barthelmess S., Wiemann S., Bieg M., Assie G., Bertherat J., Schaefer I.M., Otto C. (2014). Aberrant DNA hypermethylation of SDHC: A novel mechanism of tumor development in Carney triad. Endocr. Relat. Cancer.

[18] Letouze E., Martinelli C., Loriot C., Burnichon N., Abermil N., Ottolenghi C., Janin M., Menara M., Nguyen A.T., Benit P. (2013). SDH mutations establish a hypermethylator phenotype in paraganglioma. Cancer Cell.

